# Multicultural personality traits of Chinese university students and their effects on the psychological adjustment in the aftermath of COVID-19 in Shanghai: a scale validation

**DOI:** 10.3389/fpsyt.2024.1363809

**Published:** 2024-03-11

**Authors:** Hanzhi Fu, Muhammad Umar Nadeem, Steve J. Kulich

**Affiliations:** SISU Intercultural Institute (SII), Shanghai International Studies University (SISU), Shanghai, China

**Keywords:** COVID-19, China, students, universities, empathy, psychological adjustment

## Abstract

**Objective:**

This study aims to explore Chinese university students’ multicultural personalities and examine how they predict the psychological adjustment of students in Shanghai. In addition, the validation of Multicultural Personality Questionnaire Short Form (MPQ-SF) scale developed to assess the multicultural personality traits of individuals is also aimed in Chinese context. Data were collected after the psychological stresses from restrictions imposed by COVID-19 in China that influenced life adjustments for nearly three years.

**Method:**

A total of 1,099 university students participated in this multi-stage study. First, the Chinese version of MPQ-SF (MPQ-SF-C) was developed and validated. The impact of MPQ-SF-C dimensions was then tested through path analysis to establish the effects of Chinese university students’ multicultural personality traits on their psychological adjustment using the Schwartz Outcome Scale (SOS-10).

**Results:**

The MPQ-SF-C yielded a five-factor solution which accounted for 60.14% of the common variance. The findings indicated that cultural empathy (β = 0.23, p < 0.05), certainty seeking (β = 0.13, p < 0.05), open-mindedness (β = 0.48, p < 0.05), and emotional stability (β = 0.24, p < 0.05) had significant influences on adjustment. Only flexibility was found to have a statistically insignificant impact on adjustment at this time in this context. MPQ-SF-C and SOS-10 scales represented very good psychometric properties in terms of their reliability and validity.

**Conclusion:**

The MPQ-SF-C shows good psychometric properties and appropriateness for evaluating multicultural personalities in Chinese contexts. The multicultural personality characteristics of university students using this scale well predicted their psychological adjustment.

## 1 Introduction

The onslaught of COVID-19 and corresponding public health control measures significantly disrupted nearly every segment of the human population across the world (1–3). Among populations, the psychological adjustment of university students who were moving from their home contexts to new local cultural contexts in China was also affected when they suddenly faced unexpected conditions and were put under psychological stress, anxiety, and fear (4–6). Often their anticipated adjustment to new university city and local life there was rendered physically or psychologically impossible by travel restrictions, lock downs, required online education, and other restrictive measures (7). These preventive measures persisted three unusually long years in China until the country ended its “Zero COVID” policy in December, 2022, after which most restrictions were correspondingly lifted, and life was allowed to start returning to a “new normal”.

University students were gradually able to visit or return to campuses in person across different regions of China without intermittent COVID imposed interruptions in the new semesters of, 2023. However, the influences of COVID-19 on China have been reported to continue with some pandemic-related changes in the culture (8); and even after the lifting of restrictive measures the widespread circulation of the virus caused new losses, anxiety (9), and ongoing public health concerns throughout, 2023 (10). In this post-COVID-19 era, research in China revealed that mental health problems related to the pandemic among university students persisted and demanded serious attention (11). Because COVID imposed mental stress and illness, it is important to seek to understand whether some students have the personality traits needed to help them adjust adequately to different local conditions. Studies are needed to identify what student dispositions contribute positively to psychological adjustment, a mental health disposition, which, if not properly dealt with, can lead to stress, emotional suffering, and poor academic or communicative performances (12, 13), especially at this juncture immediately after the relatively long and abnormal period of COVID-19 restrictions in China.

China is a multicultural mosaic, consisting of highly linguistically, religiously, ethnically, culturally diverse groups in an area similar in size to continental Europe (14, 15). It is a country with a wide range of “small cultures” and the differences between them can be profound (16, 17). While existing studies tend to treat national boundaries as defining different cultures (18), it has also been shown that numerous individual-level cultural differences within a national culture can be far greater than those across different national cultures (19) and that more cultural differences can exist within a single national culture than in cross-national comparisons (20). In this case, Shanghai represents a viable site to assess university students’ psychological adjustment as it has numerous well-ranked universities that attract applicants from all over the country.

Students moving to such universities and studying in relatively new and unfamiliar cultural conditions are confronted with a wide range of psychological, sociocultural, behavioral, and academic challenges (21–25). Studies have shown that individuals respond differently regarding communication and adjustment during intercultural encounters (26–28). A significant predictor of an individual’s intercultural adjustment has been shown to be personality (29–31). In intercultural contexts, multicultural personality traits have been both theorized and shown to be salient for adjusting to a relatively new culture (32–34).

Existing studies have explored the dynamics of multicultural personalities and students’ adjustment to local conditions in varied ways (21, 35–42). Such studies are mainly focused on international students’ adjustment to local cultures (38, 40, 42) and local students’ effectiveness when travelling abroad (39, 41). Some investigations on smaller countries have shown that certain multicultural personality characteristics of students have effects on some aspects of their performance in local life (41, 42). Recent research seems promising to apply to larger contexts for assessing psychological adjustment (43, 44). However, nominal attention has been given to local students’ multicultural personality traits that represent their in-country intercultural adjustment and its impact on their psychological adjustment to different cultural contexts within a large country (45, 46). The city of Shanghai is an ideal place for such a study due to its cultural diversity; students here have more interactions with people from various cultural groups compared to other parts of China (47–49).

To assess the multicultural personality traits across cultures, the most widely used tool is the Multicultural Personality Questionnaire (MPQ) (34). It was initially developed in the Netherlands and has been applied mostly to international student participants to measure their multicultural effectiveness (34, 50). It has solid cross-cultural psychological properties and has been robustly applied in and across distinct contexts (51). The original version of the MPQ had five dimensions expressed in 91 items and every single dimension (confirmed sub-scales) is treated as one multicultural personality trait (34, 50).

Though internationally robust, an earlier Chinese translated version of the full MPQ failed to provide desired results (the factors extracted could only explain 35.59% of cumulative variance) in the cultural context of China (52). Critical examination of that study revealed that the most common problems were with item translation, item validity, and the uniqueness of Chinese culture (53) not duly considered in the design. Since then, the MPQ has not been tested in Chinese contexts.

Recently, another short-form version of the MPQ (MPQ-SF) was introduced and successfully tested by the researchers that developed and widely tested the original. The short form confirmed the same five dimensions (using eight item sub-sales for each dimension), reducing the MPQ-SF to only 40 items (28). These five personality traits include: cultural empathy, flexibility, social initiative, emotional stability, and open-mindedness (28, 34, 50). The MPQ-SF has been successfully validated in different cultures and contexts (28, 51, 54), but not yet in China. Prior studies concerning the MPQ-SF have also established the valid and salient relationships of its five factors affecting psychological adjustment of individuals in different contexts across national borders (38, 39). However, neither the MPQ-SF or a validated localized version has yet been tested or applied to students’ psychological adjustment to local cultures in China.

The current study aims to develop and verify the validity and reliability of the Chinese version of MPQ-SF (MPQ-SF-C) in a Chinese context. For this reason, the 40-item MPQ-SF was carefully translated, back-translated, checked for equivalence, then circulated to test and examine the multicultural personality traits of university-level students in China. The resulting MPQ-SF-C was then analyzed to determine which dimensions impact the psychological adjustment of university students in Shanghai in the aftermath of the COVID-19 pandemic.

## 2 Methods

### 2.1 Procedures

The sample of this study was students enrolled in three universities in Shanghai, China. A cross-sectional research design using the survey technique was adopted to collect data online via WeChat, a widely used communication application in China, from the university students recruited (by snowball sampling). The 54-item electronic survey questionnaire (requiring around 10 minutes to complete) was created with Questionnaire Star software and distributed via a QR code by invited teachers and students before classroom sessions and in WeChat chat groups. The entire data collection process took three months (September to November, 2023), and 1,099 valid questionnaires were obtained after deleting invalid questionnaires (such as those showing obvious response patterns). The sample included both undergraduate and postgraduate students enrolled in universities in Shanghai with an average age of 20.04 years (ranges: 18-32 years). Among them, 582 were male and 517 were female students. 33.0% of the students came from big cities, 26.7% were from medium or small-sized cities, and 40.3% were from towns or villages. They represent both natural and social sciences majors (see details in **Table 1**).

**Table 1:** Background of the sample.

		Frequency	Percentage
1. What is your age?	18	211	19.2
	19	345	31.4
	20	247	22.5
	21	57	5.2
	22	82	7.5
	23	96	8.7
	24	32	2.9
	25	17	1.5
	26	3	0.3
	27	3	0.3
	28	3	0.3
	29	1	0.1
	30	1	0.1
	32	1	0.1
2. What is your gender?	Male	582	53.0
	Female	517	47.0
3. What is the size of your hometown?	Big city	363	33.0
	Medium or small city	293	26.7
	Town or village	443	40.3
4. What is your major?	Social science	337	30.7
	Natural science	762	68.7

### 2.2 Measurement tools

The first section of the survey form was designed to collect standard demographic information on students’ age, gender, size of hometown, and major. The translated version of the MPQ-SF was placed in the second section, and the psychological adjustment questions (SOS-10) in the third section. All items included in the present study appeared only in Chinese. Each item was first translated by an English teacher, back translated by a native English-speaking professor proficient in Chinese, and then discussed item-by-item for confirmation with an intercultural panel of five teachers proficient in both languages following cross-cultural translation procedures (55). A 5-point Likert scale was adopted, with rankings from 1 (Totally Not Applicable) to 5 (Completely Applicable).

The original MPQ-SF (28) was adapted in the current study with the intent to develop and validate the Chinese version (MPQ-SF-C). The MPQ-SF was designed to measure multicultural personality traits in diverse populations, typically in educational environments (36, 51). Though successfully validated in multiple cultures (28, 51, 54), its application, validity, and reliability in the Chinese context needed to be examined given previous mixed results of the longer version of MPQ. The 40 items of this scale followed the 5 pre-defined dimensions (8 items each confirming the factors of cultural empathy, flexibility, social initiative, emotional stability, and open-mindedness). Typical items were “I am a good listener”, and “I start a new life easily”. The Cronbach’s α value of the international MPQ-SF scale was 0.77, and that of each dimension ranged between 0.72 and 0.82 (28).

The current study aims to validate a version of MPQ-SF in China (MPQ-SF-C) before assessing its impact on university students’ adjustment to new cultural contexts. The Cronbach’s α of the MPQ-SF-C developed in this study was 0.89. Students’ psychological adjustment was then measured by the 10 items of Schwartz Outcome Scale (SOS-10) (56). Typical items in the SOS-10 were “I feel hopeful about my future”, and “I have peace of mind”. In previous research, this scale reflected good internal consistency value (α = 0.96) (56). In this current Chinese study, this unidimensional scale also measured psychological adjustment with a satisfactory Cronbach’s α of 0.89.

### 2.3 Statistical analysis

Statistical analyses in this study were performed through SPSS 26.0 and AMOS 23.0. First, descriptive statistical analyses of MPQ-SF items were carried out using t-tests as well as skewness and kurtosis values to ensure that items were well discriminated and normally distributed. Second, exploratory factor analysis (EFA) using principal component analysis (PCA) and confirmatory factor analysis (CFA) were performed successively on two subsamples (generated by randomly dividing the data into two parts) to determine the structure of the MPQ-SF-C. Third, reliability and validity of MPQ-SF-C and SOS-10 were examined via calculating the values of Cronbach’s alpha and CFA. Fourth, path analysis was conducted testing whether multicultural personality traits predicted psychological adjustment. Lastly, the relationships between demographic information and MPQ-SF-C were also explored using analysis of variance (ANOVA). The level of significance of p < 0.001, p < 0.01, and p < 0.05 were set for all statistical analyses.

## 3 Results

### 3.1 Item analysis of MPQ-SF

Item analysis was carried out examining the relationship between each item and the whole MPQ-SF. Subjects were ranked according to the total scores they gave in descending order. The top 27% of them were put into the high subgroup while the lowest 27% into the low subgroup. The scores of each item in the two groups were then subjected to independent samples t-tests. Results indicated that scores of each item in the high subgroup were significantly higher than the low subgroup (p < 0.05). Normality of the data was supported by skewness and kurtosis values of all items: all falling within the range between ±3.0 and ±8.0 (57). Hence, all items in MPQ-SF were well discriminated and the data normality was ensured. Therefore, items were all kept for further analyses.

### 3.2 Factor analyses of MPQ-SF-C

The sample was divided randomly into two subsamples (549 and 550 participants respectively). EFA was performed on the first subsample using PCA. The results indicated that both the KMO (0.896) and Bartlett test of sphericity (χ2 = 9886.44; df = 780; p < 0.001) were good, suggesting that the sample was suitable for factor analysis. In PCA, eigenvalues greater than one and the observation of a scree plot were used to determine the number of common factors being extracted. The first round of EFA extracted 7 factors. This process was repeated several times after items with loadings less than 0.50 (58), those cross-loaded on two factors with loadings above 0.40 (59), and any factor with fewer than three items were removed (60). The last round of EFA retained 6 factors of 31 items. A further parallel analysis suggested the retaining of 5 factors (27 items). This solution was then subjected to CFA on the second subsample. CFA resulted in the removal of one more item due to factor loading smaller than 0.50, leading to a 26-item model accounting for 60.14% of cumulative variance contribution. This model was used to form the validated 26-item MPQ-SF-C.

Compared with the original MPQ-SF, the social initiative factor was not confirmed and removed. However, in MPQ-SF-C a new factor was added, which was dubbed as “certainty seeking” given its content, such that the new instrument contains the following 5 dimensions (**Table 2**): cultural empathy (8 items for assessing the individual’s ability to empathize with culturally distinct others), flexibility (4 items measuring the individual’s extent of freedom in changing behavior patterns), certainty seeking (3 items from the original MPQ-SF’s flexibility subscale, but renamed as a subscale based on evaluating the individual’s tendency to seek stability and certainty), emotional stability (5 negatively worded items measuring whether the individual can stay calm in challenging situations), and open-mindedness (6 items assessing the attitude to stay open and unbiased when faced with cultural differences).

**Table 2:** MPQ-SF-C and SOS-10.

Dimension/Item	Loading
Cultural empathy	
1. I pay attention to the emotions of others.	.73
2. I am a good listener.	.69
3. I sense when others get irritated.	.69
4. I enjoy getting to know others profoundly.	.66
5. I enjoy other people’s stories.	.62
6. I notice when someone is in trouble.	.68
7. I sympathize with others.	.63
8. I set others at ease.	.57
Flexibility	
1. I work according to strict rules.	.83
2. I work according to plan.	.87
3. I work according to strict scheme.	.89
4. I look for regularity in life.	.67
Certainty seeking	
1. I want predictability.	.65
2. I function best in a familiar setting.	.65
3. I have fixed habits.	.64
Emotional stability	
1. Sometimes, I am worried.	.72
2. Sometimes, I am nervous.	.73
3. Sometimes, I feel lonely.	.67
4. Sometimes, I feel insecure.	.73
5. Sometimes, I am under pressure.	.75
Open-mindedness	
1. I try various approaches.	.69
2. I look for new ways to attain my goal.	.71
3. I start a new life easily.	.67
4. I like to imagine solutions to problems.	.66
5. I seek people from different backgrounds.	.63
6. I have broad range of interests.	.68
Adjustment	
1. Given my current physical condition, I am satisfied with what I can do.	.61
2. I have confidence in my ability to sustain important relationships.	.69
3. I feel hopeful about my future.	.71
4. I am often interested and excited about things in my life.	.67
5. I am able to have fun.	.66
6. I am generally satisfied with my psychological health.	.70
7. I am able to forgive myself for my failures.	.60
8. My life is progressing according to my expectations.	.70
9. I am able to handle conflicts with others.	.69
10. I have peace of mind.	.68

The new instrument exhibited good model fit, as χ^2^/df (2.45) was smaller than 5, GFI (0.91), NFI (0.90) and CFI (0.93) were equal or higher than 0.90, and RMSEA (0.051) was lower than 0.08 (61). All standardized loadings were above 0.50 and CR of each dimension was 0.86, 0.89, 0.66, 0.85, and 0.84 respectively, all above the cut-off value of 0.60, confirming composite reliability and convergent validity (62). The square root of AVE for each dimension was larger than their correlation coefficients with other dimensions (**Table 3**) showing good discriminant validity (57). Therefore, MPQ-SF-C exhibited good structural validity and psychometric properties.

**Table 3:** Discriminant validity of MPQ-SF-C.

	1	2	3	4	5
Cultural empathy	**0.66**				
Flexibility	0.15***	**0.82**			
Certainty seeking	0.26***	0.30***	**0.63**		
Emotional stability	0.17***	0.01***	0.21***	**0.73**	
Open-mindedness	0.25***	0.13***	0.15***	0.05***	**0.68**

***ρ < 0.001; the square root of AVEs is shown in bold.

### 3.3 Reliability and validity of MPQ-SF-C and SOS-10

The reliability and validity of MPQ-SF-C and SOS-10 were confirmed before the regression analysis. Cronbach’s alpha values were evaluated for the determination of reliability and CFA values were assessed for the attainment of validity. The results revealed that α values for SOS-10 and MPQ-SF-C were similar (0.89). In terms of MPQ-SF-C subscales, the value of α was 0.86 for cultural empathy, 0.88 for flexibility, 0.69 for certainty seeking, which was slightly low but still considered adequate (63), 0.84 for emotional stability, and 0.84 for open-mindedness. The reliability values were better than those obtained in other versions of MPQ-SF, such as the retested original English version (51) in which α values ranged from 0.70 to 0.84, and the Spanish version (54) in which the values of α fell between 0.54 and 0.74. CFA values of both scales (MPQ-SF-C and SOS-10) as well as for their sub-scales loaded well in their corresponding measures and meet the minimal threshold of retainment. Item details and their loadings are listed in **Table 2**. Based on the confirmation of reliability and validity, the data of this study appeared valid enough to be considered for further testing.

### 3.4 Path analysis

**Figure 1:**
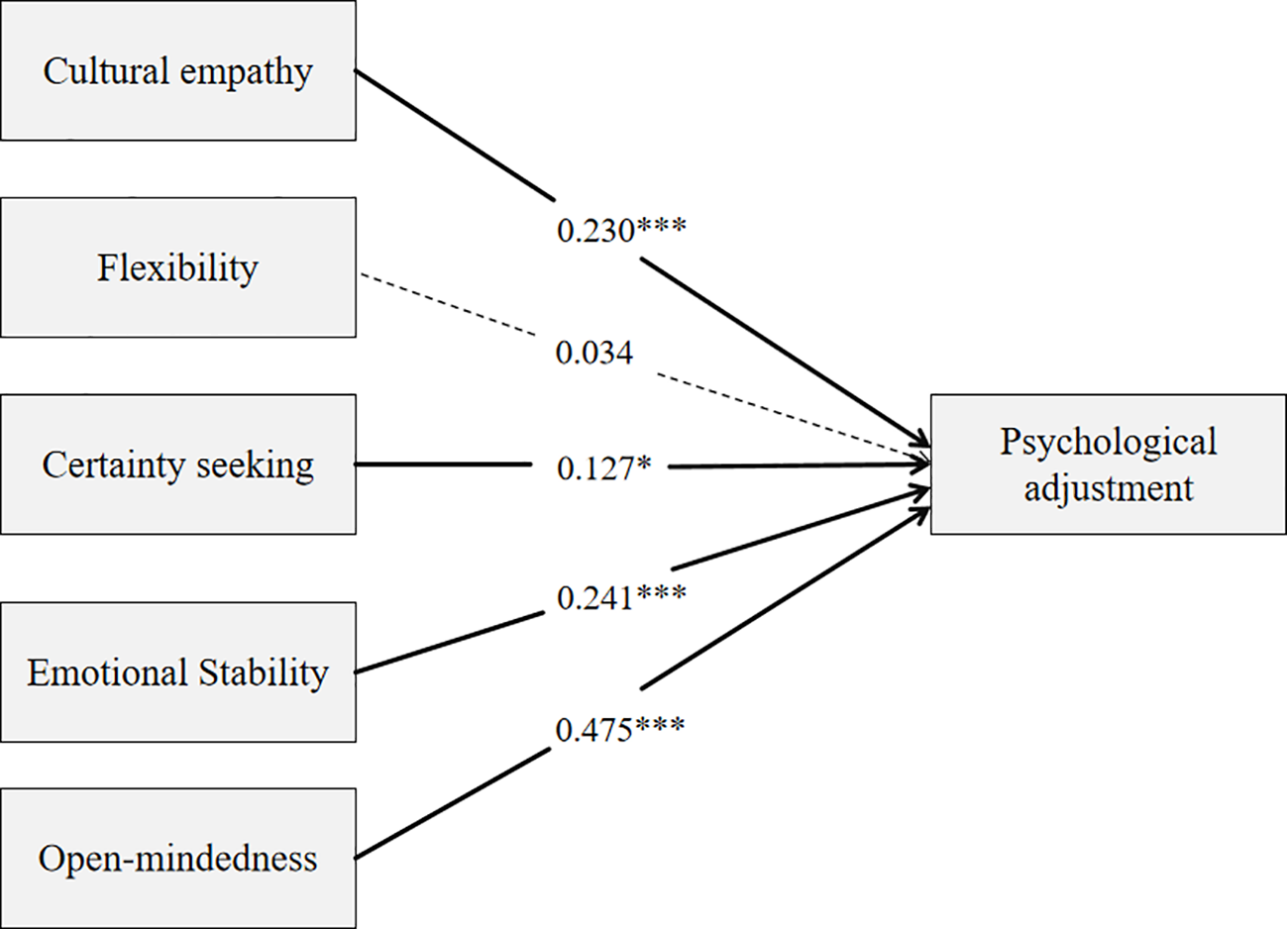
Path model of the study.

A path analysis through the structure equation modeling (SEM) was employed to analyze the influence of every single multicultural personality trait on psychological adjustment. The findings revealed that cultural empathy (β = 0.23, p < 0.05), certainty seeking (β = 0.13, p < 0.05), emotional stability (β = 0.24, p < 0.05) and open-mindedness (β = 0.48, p < 0.05) had a positive significant influence on adjustment. However, flexibility (β = 0.03, p > 0.05) had a statistically insignificant impact on the students’ adjustment (**Figure 1**). Based on the findings of this current study, the direct effect of every proposed antecedent from MPQ-SF-C was established on adjustment (**Table 4**) expect flexibility.

**Table 4:** Path analysis.

			β	S.E.	ρ	Status
Cultural empathy	➔	Adjustment	0.230	0.046	***	Accepted
Flexibility	➔	Adjustment	0.034	0.031	0.269	Rejected
Certainty seeking	➔	Adjustment	0.127	0.049	0.010	Accepted
Emotional stability	➔	Adjustment	0.241	0.028	***	Accepted
Open-mindedness	➔	Adjustment	0.475	0.041	***	Accepted

***ρ < 0.001.

### 3.5 MPQ-SF-C and demographic information

Since the direct effects of Chinese university students’ multicultural personality traits on adjustment were established, it was also critical to examine what demographic characteristics were related to their multicultural personality traits, which might have further influenced their adjustment. ANOVA was employed to compare gender, age, and size of hometown with MPQ-SF-C. Results showed that university students’ age was not statistically significant on any dimension in MPQ-SF-C. The sub-categories of gender had a statistically significant difference in reporting emotional stability (F = 9.38, p < 0.05). The sub-domains of size of hometown had a statistically significant difference regarding cultural empathy (F = 3.54, p < 0.05), open-mindedness, (F = 7.71, p < 0.05), and the complete MPQ-SF-C scale (F = 3.80, p < 0.05).

## 4 Discussion

The present study aimed to identify Chinese university students’ multicultural personality characteristics and examined how they influenced psychological adjustment to life in a distinct region of this large country immediately after several years of limitations due to COVID-19 and related restrictions. As an important predictor of individuals’ adjustment in a wide range of contexts, personality (29–31) and specifically, in cases of intercultural encounters, multicultural personalities (32, 33) have been employed by former researchers to explore their relationships with students’ adjustment to local conditions in many ways (34, 36, 38, 39, 41, 42).

As noted, literature in this area has mostly focused on cross-country adaptation of international students and that of local students travelling abroad. Although a limited number of studies in smaller countries have revealed that certain multicultural personality traits were related to some aspects of students’ performances in local conditions (41, 42), not enough attention has been given to such traits and their relationships with students’ adjustments across cultural regions in a large country. This study aimed to bridge such a gap by adapting and validating the latest version of the robust MPQ measuring multicultural personalities (51) in a Chinese context and examining how its variables predicted university students’ adjustment in the aftermath of COVID-19 in China when they could finally engage in a new local context with fewer pandemic-related psychological challenges and physical restrictions.

To develop and validate the MPQ-SF-C, the original MPQ-SF items were translated into Chinese, back-translated, and carefully discussed to ensure both content validity and the semantic consistency. After a sample of 1,099 university students in Shanghai was collected, items were analyzed via t-tests, skewness, and kurtosis values to ensure that every item was well discriminated and normally distributed. Several rounds of EFA and CFA procedures yielded a MPQ-SF-C comprising 26 items divided into 5 dimensions. Appropriate tests revealed good model fit, validity, and reliability of this new scale. 4 dimensions of MPQ-SF-C maintained the same names as 4 MPQ-SF dimensions because items confirmed in these new dimensions were consistent with corresponding ones in the original MPQ-SF. One dimension failed in this Chinese context (social initiative), and one new dimension (3 items) was separated from the original flexibility dimension and renamed certainty seeking.

It is not uncommon for scale dimensions to change in cross-cultural validation (64, 65). The new dimension that emerged was reflected in the initial MPQ design (34, 50) but suggests that the Chinese view flexibility differently. The Chinese are culturally taught to be flexible but tend to seek certainty in difficult situations (66, 67). The certainty seeking orientation might have been accentuated due to the uncertain period just experienced through the pandemic, or it might also be explained by the uncertain economic prospects brought about by a relatively long and costly period of COVID-19 impact in China (68).

In addition, the social initiative factor of MPQ-SF was not confirmed in MPQ-SF-C. That is probably because the Chinese traditionally refrain from taking direct initiatives in social contacts; they are generally regarded as preferring a high-context communication style that features restrained ways of making social contact (69). The Chinese also generally make clear distractions between ingroup and outgroup members, usually warmer and more interactive with ingroup members but relatively indifferent towards or refraining from interacting with those outside their circle (52).

After the MPQ-SF-C was developed and validated, a multivariate regression analysis was performed to examine whether or how Chinese university students’ multicultural personalities had effects on their psychological adjustment. Former studies in cross-country settings showed that emotional stability, social initiative, and cultural empathy had direct effects on students’ psychological adjustment to local conditions, whereas flexibility and open-mindedness either had indirect effects on psychological adjustment or did not have effects at all (38, 39). The current research partially confirmed their findings, within a large country, by also establishing direct effects of emotional stability and cultural empathy on psychological adjustment. Different from prior findings, this study also revealed that open-mindedness had a significant influence on adjustment. The newly named variable certainty seeking, confirmed in this Chinese context, also influenced adjustment. These findings suggested that, in the post-COVID-19 period, an individual’s multicultural personality traits can help predict psychological adjustment to new local conditions. Studies of intercultural encounters of this kind within large countries should be given more attention to.

The study further examined the influence of demographic variables on multicultural personality characteristics. Gender was statistically significant on emotional stability, suggesting men were more likely to stay stable emotionally in intercultural contexts. Open-mindedness and cultural empathy were related to hometown size, suggesting that individuals from cities were more likely to be open-minded and empathize with those from culturally different backgrounds.

In summary, COVID-19 related psychological depression, stress, anxiety (70), fear, and restrictions prevented university students all over China from opportunities to adjust to new locations for almost three years. This research explored the multicultural personality features of Chinese university students. Validating and testing the new MPQ-SF-C allowed examination of what multicultural personality traits helped them adjust psychologically after they were finally able to leave the cultural contexts of their home for university studies in a major city after the lifting of various COVID pandemic restrictions.

### 4.1 Limitations

Though this study developed and validated the first Chinese version of the MPQ-SF on a university student sample, further testing of this new instrument should continue among other populations. This research could be limited by varied hidden latent variables that were not directly considered, such as cultural value and social support influences which might have influenced the results. Thus, future studies should probe into factors of these types to examine adjustment of individuals more broadly.

## 5 Conclusion

The aim of this study was to examine the characteristics of Chinese students’ multicultural personality and their effects on students’ post-COVID-19 psychological adjustment to local conditions when they could finally be fully exposed to local life in a different urban context. Unlike findings of the previous study, the new Chinese language instrument developed by this research, MPQ-SF-C, demonstrated excellent validity and reliability. It also showed that different cultural contexts might construe similar items with different associations (such that Chinese responses did not confirm the domain of social initiative, and also considered flexibility in narrower terms as seeking certainty). Four out of five dimensions from MPQ-SF-C predicted students’ psychological adjustment, which not only partially confirmed those of previous studies but also extended this line of research to cross-cultural contexts within a large country for the first time. Additionally, gender and the size of hometown were shown to be related to students’ multicultural personality that might have further influenced their adjustment.

## Data availability statement

The raw data supporting the conclusions of this article will be made available by the authors, without undue reservation.

## Ethics statement

The studies involving humans were approved by Institutional Ethics Review Committee of the SISU Intercultural Institute (SII), Shanghai International Studies University (SISU), China (2023-SII/IRB-0801). The studies were conducted in accordance with the local legislation and institutional requirements. The participants provided their written informed consent to participate in this study.

## Author contributions

HF: Conceptualization, Data curation, Formal analysis, Investigation, Methodology, Validation, Writing – original draft. MN: Conceptualization, Investigation, Methodology, Project administration, Resources, Supervision, Validation, Writing – review & editing. SK: Conceptualization, Funding acquisition, Investigation, Methodology, Project administration, Supervision, Validation, Writing – review & editing.
